# Choroidal thickness in children with type 1 diabetes depending on the pubertal status and metabolic parameters analyzed by optical coherence tomography

**DOI:** 10.1038/s41598-021-97794-3

**Published:** 2021-10-04

**Authors:** Wysocka-Mincewicz Marta, Olechowski Andrzej, Baszyńska-Wilk Marta, Byczyńska Aleksandra, Gołębiewska Joanna

**Affiliations:** 1 The Department of Endocrinology and Diabetology, Children’s Memorial Health Institute, Warsaw, Poland; 2 The Department of Ophthalmology, Children’s Memorial Health Institute, Warsaw, Poland; 3 The Ophthalmology Department, James Cook University Hospital, Middlesbrough, UK; 4 The Faculty of Medicine, Lazarski University, Warsaw, Poland

**Keywords:** Endocrinology, Health care, Medical research, Risk factors

## Abstract

To assess choroidal thickness (CT) in children with type 1diabetes (T1D) regarding their pubertal status and seek for factors influencing this parameter, using optical coherence tomography. Material and methods: 333 eyes out of 167 children with T1D without symptoms of diabetic retinopathy (mean age 12.81 ± 3.63 years, diabetes duration 4.59 ± 3.71 years) were enrolled. CT in all quadrants was evaluated. The studied population was divided into three groups: prepubertal, pubertal and postpubertal. The multivariate regression model was carried out using all metabolic parameter and then it was built using only the significant ones. Results: Significant differences in CT between males and females, except nasal and superior quadrants were observed. We revealed significant differences in CT between the three independent groups (Chi-square 18.6, *p* < 0.0001). In the statistically significant multiple regression model (R = 0.9, R^2^ = 0.82, *p* < 0.0000), the serum level of free thyroxine, triiodothyronine, total hemoglobin, uric acid, low- and high-density cholesterol, daily insulin dose per kilogram, weight and level of vitamin D were significant. Conclusion: In our studied group CT increases during puberty. Metabolic parameters such as cholesterol, uric acid, thyroid hormones, and hemoglobin concentration even within the normal range, significantly influence the CT, and these factors likely affect other blood vessels in the body.

## Introduction

The choroid plays important role in the vision process, supplying the external retina with oxygen and nutrients. The assessment of the choroid has long been of huge interest, because systemic diseases affect the choroid due to the rich network of vessels. Histological studies show loss of choriocapillaries in patients with type one diabetes (T1D), which results in reduced choroidal blood flow, retinal tissue hypoxia as well as retinal pigment epithelium and photoreceptor dysfunction and death^1^. Choroidal vasculopathy plays an important role in the pathogenesis of diabetic retinopathy^2–4^. Optical coherence tomography (OCT) proved to be a breakthrough in choroidal imaging and choroidal thickness (CT) measured in OCT is considered as a putative measure of choroidal blood flow.

The incidence of type 1 diabetes mellitus (T1D) in children is still increasing^5^. It has been observed that decreased CT is associated with diabetic retinopathy development^1^. The other studies have revealed that in children during the early period of diabetes choroid becomes significantly thicker^6,7^. The influence of pubertal status on CT was assessed by Hansen et al. in the CCC2000 Eye Study^8^ but to our knowledge no study was directly focused on puberty influence on children with T1D. Our previous results showed no significant difference in CT in the group of diabetic children compared to the control group, but the results regarding the pubertal status were not considered^9^. The aim of the study was to assess CT in children with T1D regarding their pubertal status and seek the associations between CT and metabolic parameters.

## Material and methods

**Figure 1:**
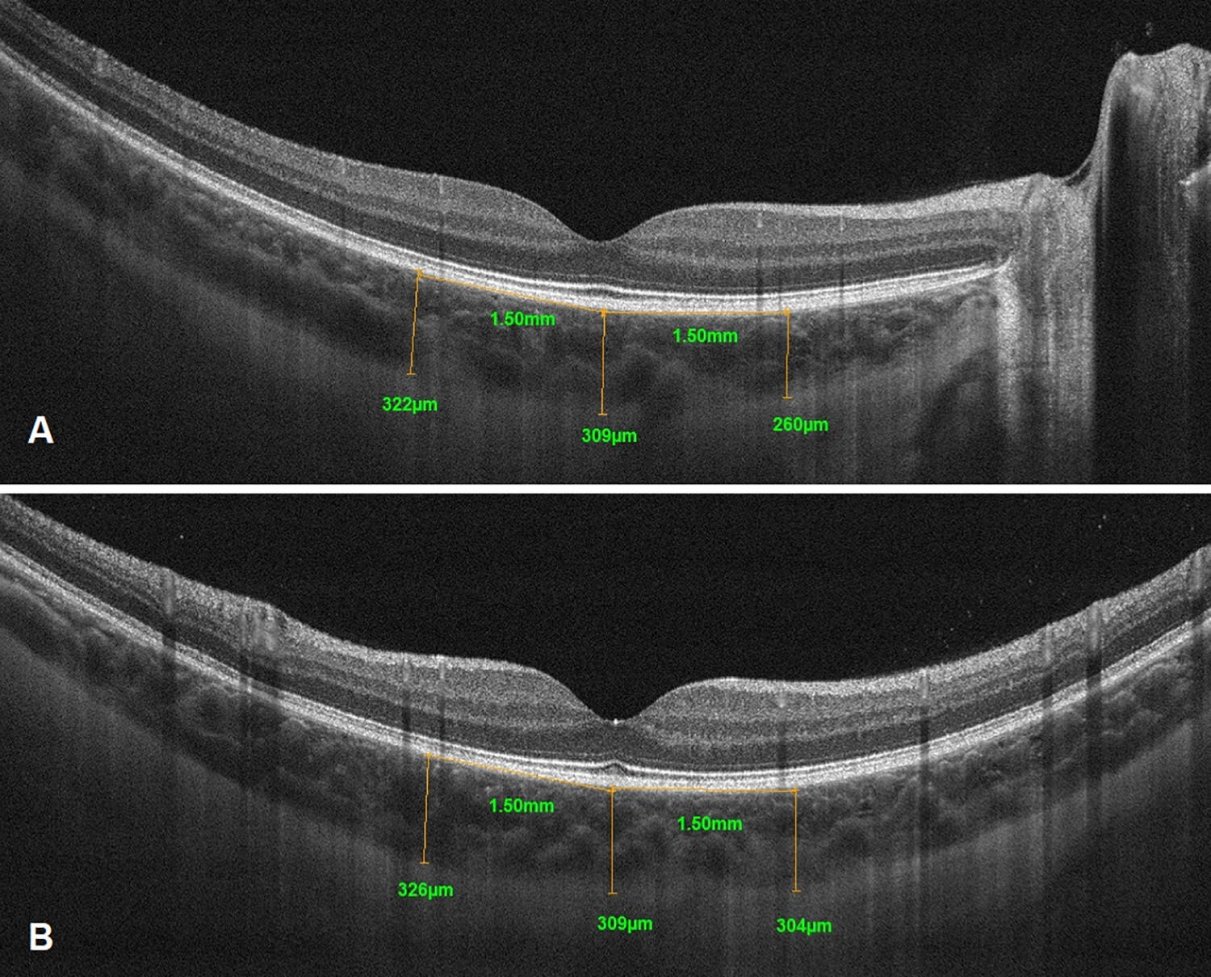
Representative OCT images—cross line scan horizonal (**A**) and vertical (**B**) reflecting measurement of subfoveal choroidal thickness and choroidal thickness in four macular quadrants.

This cross-sectional study is one in a series of studies in population of 175 children with T1D, without signs of DR. The diagnosis of T1D was based on the criteria of the International Society for Paediatric and Adolescent Diabetes (ISPAD), and all patients needed insulin treatment. The study was approved by the Institutional Review Board and followed the tenets of the Declaration of Helsinki. After explanation of the nature and scheme of the study, a written informed consent was obtained from the patient’s parent or legal guardian (all participants were under 18**).** Exclusion criteria were history of prematurity and other concomitant retinal pathologies, such as hereditary retinal dystrophies, vitreoretinal diseases, as well as uveitis, glaucoma, and high refractive error (spheric equivalent >  + / − 3.00 diopters). Every patient underwent a complete ocular examination, including best-corrected visual acuity (BVCA), slit lamp biomicroscopy, dilated fundus examination, and color fundus photography. Clinical data recorded for each subject included age, age at onset, duration of diabetes, weight, height, body mass index (BMI), total hemoglobin (Hb), level of cholesterol (TC), low-denisty lipoprotein cholesterol (LDL-C) and high-density lipoprotein cholesterol (HDL-C), triglicerides, serum creatinine, thyrotropin (TSH), free thyroxine (fT4), free triiodothyronine (fT3), uric acid (UA), level of vitamin D3 (25OHD3), pH at the moment of diagnosis, systolic and diastolic blood pressure, mean (mean value for the whole diabetes duration, minimum 4 tests per each year), and actual levels of glycated hemoglobin (HbA1C), as well as mean and actual levels of daily urine albumin excretion, mean total daily insulin dose, daily insulin dose per kilogram. The study population was divided according to their pubertal status into 3 groups: prepubertal (Tunner stage 1), pubertal (if any signs of pubertum are observed, Tunner stage 2–4) and postpubertal (Tunner stage 5). OCT was performed using a commercially available RTVue XR Avanti (Optovue, Fremont, CA, USA Version 2018.0.0.18). The imaging parameters of this OCT system are as follows: central wavelength 840 nm, bandwidth + /− 25 nm, power of light source 750 μW, axial resolution 5 μm, lateral resolution 15 μm. To obtain high-quality images of the retina and choroid, a 10-millimeter (80.000 A scans/second) cross Line scan was performed in the fovea, which is the centre of fixation in a healthy subject. This scan consists of two perpendicular scans—one horizontal and one vertical, crossing the fovea. Choroidal thickness in all patients was measured at the same points in relation to the fovea. CT was defined as the distance between the hyperreflective line corresponding to the outer boundary of the RPE and the hyperreflective line corresponding to the chorioscleral interface and was measured manually using the built-in calipers in OCT software. The measurements were obtained in the subfoveal region (central choroidal thickness – CCT) and at a distance of 1500 μm superiorly, inferiorly, nasally, and temporally from the center Fig. 1.

All measurements were performed at the same time of the day (between 9:00am and 11:00am) in all children to avoid the effect of diurnal CT variation on the results. Eyes with low–quality scans (< 6), with motion artifacts or blurred images were excluded from final analysis (17 eyes). Both eyes' results were analyzed because of the influence of metabolic parameters and interocular differences.

### Statistical analysis

Data were described by mean, and standard deviation, minimum and maximum values, and median, 25–75 percentiles. Normal distribution was checked using Shapiro–Wilk test. Differences between two groups in parameters with normal distribution were tested by t-student test, but parameters without normality of distribution were analyzed using U Mann–Whitney test. Differences between 3 independent groups by Kruskal–Wallis H test were analyzed. For the analysis of correlations between parameters multiple regression analysis were used. In the first step all collected descriptive and metabolic data were taken, then to the model were chosen only statistically significant parameters. A level *p* < 0.05 was recognized as statistically significant. Statistical analysis was performed using the STATISTICA® 13 PL (2017, StatSoft, Poland) package from https://www.statsoft.pl.

### Ethical approval

The study was conducted according to the guidelines of the Declaration of Helsinki and approved by the Ethics Committee of The Children’s Memorial Health Institute 11/KBE/2017.

### Informed consent

A written informed consent was obtained from the patient’s parent or legal guardian (all participants were under 18).

## Results

Three hundred thirty three eyes out of 167 children were finally included in the study (17 scans were excluded because of poor quality). The characteristics of the studied groups are summarized in Tables 1 and 2.

**Table 1:** Characteristics of the studied patients.

Investigative trait	Mean	SD	Median	Min	Max
Age (years)	12.81	± 3.63	13.04	4.5	18.0
Diabetes duration (years)	4.59	± 3.71	3.7	0.02	15.33
Age at onset (years)	8.22	± 3.81	7.89	1.46	17.04
Weight (kg)	47.39	± 18.22	46.00	16.50	93.5
Height (cm)	155.4	± 19.47	159.99	106.0	191.50
BMI (kg/m2)	18.79	± 3.78	17.82	11.99	32.56
HbA1c current (%)	8.3	± 1.79	7.8	5.8	14.00
HbA1c mean (%)	7.8	± 1.2	7.7	6.0	12.00
Hb (g/l)	13.88	± 1.14	13.7	11.3	17.2
Daily insulin dose per kilogram of weight (U/kg)	0.75	± 0.26	0.75	0.11	1.3
TSH (µIU/ml)	2.34	± 1.1	2.2	0.46	7.5
fT4 (ng/dl)	1.08	± 0.4	1.01	0.76	3.75
fT3 (ng/dl)	2.96	± 0.63	3.02	0.79	4.18
Cholesterol total (mg/dl)	167.47	± 31.98	166.0	102.0	304.0
LDL cholesterol (mg/dl)	86.31	± 25.0	84.0	32.0	192.0
HDL cholesterol (mg/dl)	65.08	± 16.36	64.0	30.0	121.0
Triglycerides (mg/dl)	79.86	± 45.23	67.0	27.0	342.0
Uric acid (mg/dl)	3.93	± 0.88	3.9	1.7	6.7
Serum creatinine (mg/dl)	0.63	± 0.17	0.6	0.32	1.27
250H D3 vitamin (ng/dl)	24.54	± 7.87	23.15	6.0	46.8
pH at the diabetes onset	7.31	± 0.11	7.35	6.9	7.45
Systolic pressure (mmHg)	109.3	± 10.74	109	84	140
Diastolic pressure (mmHg)	63.36	± 9.21	65	40	91

**Table 2:** Characteristic of the groups regarding pubertal status.

	Prepubertal group n = 52 girls = 24 boys = 28	Pubertal group n = 70 girls = 32 boys = 38	Postpubertal group n = 45 girls = 26 boys = 19	*p* level
Age	8.4 ± 1.6	13.1 ± 1.5	17.2 ± 0.7	*p* < 0.0000
Age at the onset (years)	5.6 ± 2.3	8.7 ± 3.4	10.5 ± 4.3	*p* < 0.0000
Diabetes duration (years)	2.7 ± 2.0	4.4 ± 3.5	6.7 ± 4.4	*p* < 0.0000
Weight (kg)	30.6 ± 12.5	48.3 ± 11.3	67.8 ± 12.5	*p* < 0.0000
Height (cm)	133.5 ± 13.3	161.8 ± 11.2	172.3 ± 9.5	*p* < 0.0000
HbA1c current (%)	7.9 ± 2.1	8.3 ± 1.8	8.6 ± 1.4	*p* < 0.0000
HbA1c mean (%)	7.9 ± 1.9	8.1 ± 1.4	8.4 ± 1.3	*p* < 0.0000

*HbA1c* glycated haemoglobin.

In the studied population we observed significant differences in CT between males and females, except nasal and superior quadrants (Table 3).

**Table 3:** CT results in females and males.

CT (μm)	Females (mean ± SD)	Males (mean ± SD)	*p* level
Central choroidal thickness	346.7 ± 97.2	322.3 ± 84	*p* < 0.02
Nasal quadrant	277.9 ± 81	266.1 ± 79.7	*p* = 0.2
Temporal quadrant	337.8 ± 80.1	317.1 ± 73.6	*p* < 0.02
Superior quadrant	332.9 ± 87.6	318.3 ± 80	*p* = 0.14
Inferior quadrant	361.6 ± 86.2	334.4 ± 85.6	*p* < 0.008

Analyzing three independent groups of children with diabetes (prepubertal, pubertal and postpubertal) we observed statistically significant differences in CT between the groups (Chi-square 18.6, *p* < 0.0001, prepubertal group vs pubertal *p* < 0.04, prepub vs postpub *p* < 0.0005, pubertal vs postpub *p* < 0.04) (Table 4). After dividing the studied population depending on the sex the statistically significant differences in CT were detected in males (Chi-square 7.3, *p* < 0.03, Kruskal–Wallis H = 9.43, *p* < 0.005, prepubertal group vs pubertal *p* = 0.1, prepub vs postpub *p* < 0.0003, pubertal vs postpub *p* < 0.05), in nasal quadrant (Chi-square 10.3, *p* < 0.005, Kruskal–Wallis H = 9.94, *p* < 0.01, prepubertal group vs pubertal p = 0.1, prepub vs postpub *p* < 0.001, pubertal vs postpub *p* < 0.01), and superior quadrant (Chi-square 5.62, *p* < 0.05, Kruskal–Wallis H = 6.84, *p* < 0.05, prepubertal group vs pubertal *p* = 0.1, prepub vs postpub *p* < 0.01, pubertal vs postpub *p* = 0.1). In the group of females only CCT had border significancy (Chi-square 12.4, *p* < 0.003, Kruskal–Wallis H = 4.4, *p* = 0.1).

**Table 4:** The differences between CT in prepubertal, pubertal and postpubertal children.

Parameter CT	Prepubertal median (min–max)	25–75 percentyl	Pubertal median (min–max)	25–75 percentyl	Postpubertal median (min–max)	25–75 percentyl
CCT	300.5 (101–764)	262.5–350	325.0 (102–585)	281–388	360.5 (166–536)	309–410
Nasal quadrant	245.0 (120–482)	208–300	261.0 (74–568)	223–321	292.0 (140–542)	238–334
Temporal quadrant	302.0 (190–615)	268–346	322.0 (118–536)	276–378	339.5 (169–546)	295–386
Superior quadrant	307.0 (157–686)	268–344	309.0 (136–533)	280.5–381.5	331.5 (149–568)	285.5–379
Inferior quadrant	329.0 (169–640)	287–378	337.5 (166–599)	293.5–396.5	351.0 (157–561)	307–415.5
**Males**						
CCT	300.0 (101–471)	248–341	322.0 (102–533)	286–357	367.0 (189–527)	297–421
Nasal quadrant	239.5 (129–424)	200–294	250.0 (74–467)	221–303	305.0 (140–542)	257–359
Temporal quadrant	300.0 (190–416)	266–342	318.0 (118–509)	272–359	330.0 (170–546)	295–381
Superior quadrant	300.5 (157–442)	262–332	300.5 (136–533)	280–368	353.5 (149–534)	285–388
Inferior quadrant	324.0 (169–498)	278–367	326.0 (166–553)	273–388	350.0 (188–486)	287–416
**Females**						
CCT	307.0 (101–764)	271–381	336.0 (102–585)	273–412	358.0 (166–536)	329–402
Nasal quadrant	251.0 (120–482)	210–302	284.0 (138–568)	236–334	278.0 (148–483)	238–316
Temporal quadrant	310.0 (213–615)	274–356	339.0 (160–536)	281–406	344.5 (169–515)	292–394
Superior quadrant	314.0 (213–686)	272–350	326.5 (170–512)	283–406	320.0 (177–568)	286–373
Inferior quadrant	331.0 (219–640)	306–408	363.0 (217–599)	312–417	351.0 (157–561)	317–412

In the analysis of parameters influencing the CT we revealed a statistically significant multiple regression model (R = 0,9, R^2^ = 0.82, *p* < 0.0000), which confirmed that serum creatinine (β = − 119.4, *p* < 0.03), level of fT4 (β = − 79.109, *p* < 0.001), fT3 (β = − 70.695, *p* < 0.002), total Hb level (β = 48.8, *p* < 0.00005), uric acid level (β = − 41.676, *p* < 0.02), LDL-C (β = 1.309, *p* < 0.003), HDL-C (β = 1.066, *p* < 0.03), daily insulin dose per kilogram (β = 57.465, *p* < 0.01), weight (β = 1.734, *p* < 0.001), and level of vitamin D (β = − 2.132, *p* < 0.02), were significant. There was also a correlation, but not a significant one with TSH (β = − 1.82, *p* = 0.06), diabetes duration (β = − 7.558, *p* = 0.08), and pH at the onset of diabetes (β = − 74.333, *p* = 0.25).

## Discussion

Diabetes mellitus during its course has a range of potential complications related to its damaging effect on the blood vessels. One of the most prevalent and influencing qualities of life is diabetic retinopathy (DR). Most of the previous studies revealed choroidal thinning associated with developing DR, but the exact mechanism of this effect remains unknown and probably may be associated with decreased blood flow through impaired choroidal vessels and increased vascular resistance leading to hypoxia^6,7^.

The previous reports in children population found no significant differences in CCT between the T1D patients and the healthy controls^9,10^, but there are a few studies focused on the peripapillary choroid. In the study of 103 pediatric patients Ermerak et al. observed significant differences in the superonasal, nasal, inferonasal and inferior sectors of the peripapillary choroid^11^. Also, Vujosevic et al. revealed a significant decrease in peripapillary CT with the increase of severity in diabetic retinopathy in adults, but without any differences in the group without DR^12^. The studies on the influence of the pubertal status on the course of the disease focused mainly on the retinopathy. The results revealed that adolescents may be linked to a higher risk of DR than young people with no history of diabetes during puberty^13–18^. These changes are most likely caused by hormonal alterations during puberty like an increased growth hormone, a decreased insulin-like growth factor 1 (IGF-1), an increased free androgen index (FAI) higher in diabetics than in control population, lower sex hormone-binding globulin (SHBG), higher body mass index (BMI) in addition to increased an HbA1c level in this period. In this study we compared choroidal thickness in children with T1D with a different pubertal status. As we have established CT increases with puberty, but we are aware that it is strongly dependent on the age but on not diabetes itself. In our analysis diabetes duration exists in the model, but has no statistically significant influence. Increased insulin resistance in patients with T1D (receiving insulin) could be determined directly by using only an insulin clamp, but clinically it is indirectly measured by a parameter of a daily dose of insulin per kilogram. In our analysis this parameter was one of the factors influencing the CT most. It should be emphasized that almost all the relevant parameters in the multivariate model are elements of the metabolic syndrome (MS) (LDL -C, HDL-C, and uric acid), including the weight, although this parameter has not been significant in the previous studies^9^. The fasting hyperinsulinemia in healthy individuals, independently of the age and arterial hypertension, has a negative impact on the retinal vessel status^19^. Insulin is an important signal for nitric oxide release from vascular endothelial cells, resulting in vasodilation and reduced vascular resistance, which reduces blood pressure^20^. Estimating the prevalence of DR in 2551 participants with the metabolic syndrome, the higher number of MS components increased the risk of DR (adjusted to HbA1c, age, sex, duration of diabetes)^21^. The other studies found that the presence of hyperinsulinemia and dyslipidemia in type 2 diabetes was associated with the onset of microvascular complications^22,23^, similarly to our results.

The uric acid has been recognized as a risk factor for diabetes vascular complications for many years, because of its proatherogenic properties, due to the activation of endothelial cells and platelets and increased platelet adhesiveness^24^. The increase of serum uric acid level due to the inflammatory process results in the progression of diabetic vascular complication, which causes vascular leakage and increased macular thickness. Xia et al. demonstrated that uric acid might be a risk factor for diabetic retinopathy^25^. In contrast to them Vinuthinee^24^ showed no correlations between serum uric acid level and retinal nerve fibre layer thickness (RNFL) and macular thickness between diabetic patients without DR and with non-proliferative diabetic retinopathy. Krizova et al. conducted studies on vitreous concentration of uric acid, and its dependences on vascular-endothelial growth factor (VEGF), influencing the severity of DR^26^. All these studies were conducted in the groups of patients with T2D, with a high level of serum uric acid. In our study all the patients had a normal value of this parameter, but its trends had a strong negative correlation with changes in CT, which may suggest that even a slight increase in a uric acid level may affect the proper growth and maturity of the choroid during puberty. This finding needs further investigation and it may be advisable to recommend early treatment in adolescents with T1D in order to achieve lower urinary acid levels. It should be highlighted that in our model the level of total hemoglobin was significantly important, which could confirm the importance of the normal blood count and proper oxygen and nutrients resourcing. Anemia accelerates the progression of DR by exacerbation of retinal hypoxia, which leads to production of growth factors such as a vascular endothelial growth factor (VEGF)^27^, a strong stimulant of neovascularization, also increasing vascular permeability and retinal exudates. In the ETDRS study, low hematocrit was found as an independent risk factor for high-risk proliferative DR and visual impairment^28^. Anemia is also an important risk factor for clinically significant macular edema^29^. Yumusak et al.^30^ detected that the group of females with anemia had significantly lower values of CCT than the control group and in the correlation analysis there was negative regression. Our study group had a normal hemoglobin level, similarly to the other biochemic parameters. The choroid is affected by blood pressure and intraocular pressure due to an autoregulatory mechanism^31^. Although we analyzed the influence of systolic and diastolic pressure, these parameters were not included in our regression model. The other factors statistically influencing the thickness of the choroid are thyroid hormones, which was confirmed in our previous study, comparing the results of children with T1D to those who had coexisted autoimmune thyroiditis^32^. In the current analysis thyroid stimulating hormone (TSH) exists in the model of regression but was not significant. On the other hand, both free hormones, thyroxine and triiodothyronine were significant and had a strong impact in the model. Only Rodacki et al. revealed the influence of proper TSH level (range 0.4–2.5 mU/l) on lowering the risk of DR in the T1D population (children were 34% of the studied group), independently of glycemic control and duration of diabetes^33^.

In our multivariate model, the levels of LDL-C and HDL-C turned out to be important factors. The influence of dyslipidemia on the risk of DR was studied in the CURES Eye Study^34^, which showed that total cholesterol, HDL-C and serum triglycerides were associated with DR. Meta-analysis of case–control studies revealed that mean levels of serum total cholesterol, LDL-C, and triglicerids were significantly higher in patients with diabetic macular edema (DME) compared to the subjects without DME^35^.

Hansen et al.^8^ examined a large group of healthy adolescents aged 11–16 and OCT based measurement of CT revealed its increase during puberty. We noticed a similar pattern of a CT increasing in all the subgroups of our diabetic patients, which might suggest that T1D does not affect CT during this period, probably due to quite good metabolic control in the studied group^8,36^. In our group the results of CT were similar to the ones previously described and the CT was the thickest in the subfoveal area, and thinner in the nasal and temporal quadrants^37–39^. In our analysis, according to the previous research^9^, CT did not depend on the age, the age of the diabetes onset or on diabetes duration. Kinoshita et al.^40^ observed in the adult group that CT depends on the age of the subjects, axial length, sex, and stage of DR. We studied a population of children without any DR symptoms, therefore we could not show such differences, but we also observed gender differences in CT in temporal and inferior quadrants.

The last factor influencing the CT in our multivariate model, having the strongest impact was the serum creatinine level. The Sankara Nethralaya DR Epidemiology and Molecular Genetic Study (SN-DREAMS) revealed that microalbuminuria increases the risk of DR twice, and the presence of macroalbuminuria increases the risk almost 6 times^41^. In the CURES study, the risk of nephropathy was found to be significantly higher in sight-threatening DR group compared to the group without DR (odds ratio 5.3, *P* < 0.0001)^42^. Hence the assessment of the renal parameters—blood urea, serum creatinine and microalbuminuria, is important, especially if DR is present.

The study has some limitations. It was a single center study. We did not have a sufficiently large control group of healthy children with a diagnosed pubertal status to compare the data. The strength of the study was a large group of children with T1D without DR, and many studied parameters, which allowed to recognize pathways, and factors underlying the pathophysiology of choroidal abnormalities. The multivariate regression model of factors influencing CT was surprising at first, but finally turned out to be very logical and holistic. The results of the study impose strict control of biochemical parameters, lowering them even to the bottom range. So far, we do not have special recommendations in pediatric population with T1D regarding pharmacological treatment as to the upper limits of the range of hyperuricemia, hyperlipidemia, subclinical hypothyroidism, or a slightly decreased hemoglobin level. However, in adult population with diabetes recommendation to lower LDL-C below the normal level has existed for many years. These conclusions need to be confirmed in further large prospective studies.

## Data Availability

The datasets generated during the current study are available from the corresponding author on reasonable request.
